# MetaBakery: a Singularity implementation of bioBakery tools as a skeleton application for efficient HPC deconvolution of microbiome metagenomic sequencing data to machine learning ready information

**DOI:** 10.3389/fmicb.2024.1426465

**Published:** 2024-07-30

**Authors:** Boštjan Murovec, Leon Deutsch, Damjan Osredkar, Blaž Stres

**Affiliations:** ^1^University of Ljubljana, Faculty of Electrical Engineering, Ljubljana, Slovenia; ^2^University of Ljubljana, Department of Animal Science, Biotechnical Faculty, Ljubljana, Slovenia; ^3^The NU, The Nu B.V., Leiden, Netherlands; ^4^Department of Pediatric Neurology, University Children's Hospital, University Medical Centre Ljubljana, Ljubljana, Slovenia; ^5^University of Ljubljana, Medical Faculty, Ljubljana, Slovenia; ^6^D13 Department of Catalysis and Chemical Reaction Engineering, National Institute of Chemistry, Ljubljana, Slovenia; ^7^University of Ljubljana, Faculty of Civil and Geodetic Engineering, Ljubljana, Slovenia; ^8^Department of Automation, Biocybernetics and Robotics, Jožef Stefan Institute, Ljubljana, Slovenia

**Keywords:** microbial metagenomics, bioinformatics pipeline, machine learning, human gut microbiome, sequence processing, non-communicable diseases, singularity, bioBakery

## Abstract

In this study, we present MetaBakery (http://metabakery.fe.uni-lj.si), an integrated application designed as a framework for synergistically executing the bioBakery workflow and associated utilities. MetaBakery streamlines the processing of any number of paired or unpaired fastq files, or a mixture of both, with optional compression (gzip, zip, bzip2, xz, or mixed) within a single run. MetaBakery uses programs such as KneadData (https://github.com/bioBakery/kneaddata), MetaPhlAn, HUMAnN and StrainPhlAn as well as integrated utilities and extends the original functionality of bioBakery. In particular, it includes MelonnPan for the prediction of metabolites and Mothur for calculation of microbial alpha diversity. Written in Python 3 and C++ the whole pipeline was encapsulated as Singularity container for efficient execution on various computing infrastructures, including large High-Performance Computing clusters. MetaBakery facilitates crash recovery, efficient re-execution upon parameter changes, and processing of large data sets through subset handling and is offered in three editions with bioBakery ingredients versions 4, 3 and 2 as versatile, transparent and well documented within the MetaBakery Users’ Manual (http://metabakery.fe.uni-lj.si/metabakery_manual.pdf). It provides automatic handling of command line parameters, file formats and comprehensive hierarchical storage of output to simplify navigation and debugging. MetaBakery filters out potential human contamination and excludes samples with low read counts. It calculates estimates of alpha diversity and represents a comprehensive and augmented re-implementation of the bioBakery workflow. The robustness and flexibility of the system enables efficient exploration of changing parameters and input datasets, increasing its utility for microbiome analysis. Furthermore, we have shown that the MetaBakery tool can be used in modern biostatistical and machine learning approaches including large-scale microbiome studies.

## 1 Introduction

Numerous decisions are made by health care providers in medicine on the basis of a multivariate set of descriptors estimating probability that a specific disease is present in an individual (diagnostic context) or a specific condition is going to occur in the near future (prognostic context). In the former diagnostic case the probability that a particular disease may be present is useful for directing patients for further testing or start of immediate treatment next to exclusion of certain causes of observed symptoms. In the latter prognostic context predictions can be utilized to plan therapeutic decisions based on the risk for developing medical condition within specific timeframe and to stratify participants in intervention trials (Collins et al., 2015; Moons et al., 2015). In either context, the combined information from multiple predictors observed and measured in an individual sample are utilized due to the fact that information from a single predictor is often insufficient to provide reliable estimates of diagnostic or prognostic value. Therefore multivariable models are being developed, validated with the aim to assist doctors and individuals in estimating probabilities and potentially guide their decision making (Collins et al., 2015; Moons et al., 2015).

However, recently the quality of reporting of prediction model studies was shown to be poor, therefore several initiatives such as TRIPOD (Transparent Reporting of a multivariable prediction model for Individual Prognosis Or Diagnosis Initiative) (Collins et al., 2015), SPIRIT-AI (Standard Protocol Items: Recommendations for Interventional Trials-Artificial Intelligence) (Cruz Rivera et al., 2020a,b), CONSORT-AI (Consolidated Standards of Reporting Trials-Artificial Intelligence) (Liu et al., 2020a,b) were initiated to name a few. In addition, FAIR guiding principles for research software (Findable, Accessible, Interoperable, Reusable) were introduced in 2022 (Barker et al., 2022; Loftus et al., 2022). This marked a significant milestone for the research community, acknowledging the growing importance of research software globally. These principles also established guidelines outlining minimum requirements for reporting algorithms in healthcare, emphasizing qualities such as explainability, dynamism, precision, autonomy, fairness, and reproducibility (Loftus et al., 2022).

Finally, good data management is the key leading to knowledge discovery and innovation, data integration and reuse by the community after the publication process. FAIR guiding principles for scientific data management (Wilkinson et al., 2016) put specific emphasis on enhancing the ability of machines to automatically use the data and support its reuse by the community to maximize the added value. These principles also take into consideration sharing conditional on privacy considerations (GDPR), claims of proprietary control, practical constraints, access privileges, and the quality of accompanying metadata (Boeckhout et al., 2018).

Recently, two larger scale reports were published describing fecal microbiome-based machine learning for multi-class disease diagnosis (Gupta et al., 2020; Su et al., 2022) utilizing species-level gut microbiome information layer derived metagenomics sequencing runs. Detecting early signs of disease before specific diagnostic symptoms appear is crucial, particularly using biological samples that allow detailed characterization and can be collected noninvasively and regularly. This presents a promising opportunity for developing straightforward prescreening tests to aid both doctors and individuals in decision-making. However, these connections between human health and the accompanying microbiome must be based on real-world conditions observed in the population, ensuring reliability and robustness across various human subjects, conditions, sub-populations, and other factors.

In addition to scientific research, also the industry for (human) microbiome-targeted products is faced with several challenges related to reproducibility and scientific rigor, which can impact the reliability and validity of research findings and the development of microbiome-based products. The primary challenges in microbiome research include the absence of standardized methods and protocols for sample collection, processing, sequencing, and data analysis. Variability in samples affected by host genetics, environmental factors, diet, lifestyle, and other confounding factors all add to complexity. Additionally, limited data sharing and transparency, including controlled access to organized raw data, metadata, and analysis pipelines with respective hyperparameters hinder independent validation of results and the advancement of scientific rigor in this field (Pray et al., 2013; Sinha et al., 2015; Ma et al., 2018; D’Elia et al., 2023; Ruxton et al., 2023).

Broad data sharing policies now enforce the repurposing of existing data from published studies. This serves as real-world data for discovering widely applicable principles and methodologies, generating hypotheses, and validating results. By integrating diverse large datasets from thousands of participants across numerous countries, this approach offers a holistic view at a scale that surpasses single publication datasets.

Existing methods are designed based on the strong assumption that the data with sufficient sample size and accurate and detailed metadata information is available to design groups or train models. The current metadata of a considerable number of sequencing samples is incomplete, misleading, or not publicly available (Kumar et al., 2024), which may lead to these methods being infeasible or causing bias in biomarker inference. Moreover, their intrinsic design in using known phenotype information makes them incapable of revealing new subtypes or stages of diseases (Liu et al., 2022). The taxonomic analysis alone may induce spurious biomarkers since diverse microbial communities from different patients can perform remarkably similar functional capabilities as shown before.

Identification of biomarkers at the level of taxonomy although utilizing species information does not make use of all other layers of information derived from metagenomics, namely alpha diversity, functional genes, enzymatic reactions, metabolic pathways, metabolites that hence remain unexplored. In addition, the gap between analyses of data using various generations of the same software remains underappreciated source of additional error, as textual information remains cited throughout the published literature while the underlying data re-analyses utilizing different versions of software and underlying databases may support advanced conclusions. Finally, the overall complexity of programs and the supporting databases constitutes another barrier for their deployment on high performance computing (HPC) or cloud computing. To fill this gap, we provide advances on many fronts, by (i) building a reproducible, stable, HPC ready, singularity image integrating the necessary plethora of heavy duty tools from bioBakery, mothur and MelonPann origin (Schloss et al., 2009; Segata et al., 2012; Truong et al., 2015; Pasolli et al., 2017; Franzosa et al., 2018; McIver et al., 2018; Mallick et al., 2019; Schloss, 2020; Beghini et al., 2021), (ii) analyzing previously utilized datasets (Gupta et al., 2020) in conjunction with not yet integrated datasets of clinical relevance (Youngblut and Ley, 2021), (iii) extending the analyses to novel layers of information (functional genes, enzymatic reactions, metabolic pathways, metabolites), (iv) assembling metadata from various studies, and (v) organizing the data into a complete machine learning dataset amenable for 70% of data for training and unseen 30% for validation. Finally, (vi) the meta integration of bioBakery v2, v3 and v4 versions of workflows of original publications enables anyone to back-map the mismatch between the original publications and advancement of algorithms and databases. In total, 4,976 publicly available samples pooled across multiple studies exploring 17 disease types in relation to healthy cohorts reported from 15 countries before, were analyzed. The wealth of data, rigorous analytical approach in data deconvolution and ML provide significant novel insight and actionable models for recognition of medical conditions over a large international dataset.

## 2 Materials and methods

### 2.1 Multi-study integration of human gut metagenomes

Data collection was commenced as described and detailed before (Gupta et al., 2020; Supplementary Table S1). In short, published studies with publicly available WGS metagenome data of human stool (gut microbiome) and corresponding subject metadata were included. Also, where multiple samples were taken per individual across different time-points only the baseline first or so-called baseline samples reported in the original study were utilized. To keep up with the same stringency as in the original study, studies reporting on diet or medical interventions or children (<10 years of age) were excluded, in addition to samples collected from disease controls but not marked as healthy or without diagnose assignment in the original study. The primary criteria for data selection included the number of samples, comparable sequencing depth, the quality of QC-ed sequences, and availability of corresponding metadata.

Metadata were synchronized for Healthy group across complete dataset with respect to their BMI and assigned the following categories, irrespective of their initial classification in the original studies: underweight (BMI < 18.5), overweight (BMI ≥ 25 and < 30), or obese (BMI ≥ 30). Consequently, stool metagenome data were renamed as underweight, overweight, or obese in our analysis. In addition, the .fastq files from the following additional projects were included: (i) a subset of the Flemish Gut Flora Project dataset was acquired to explore the efficiency of fecal microbiome data layers in classification of depression based on fecal metagenomic data and metadata (age, sex, BMI, BSS, RAND) of 150 subjects (*M* = 50, SD = 12,96, 38% male) – 80 with depression and 70 healthy controls (Valles-Colomer et al., 2019); (ii) samples of the PreTerm project (*n* = 24) (Deutsch et al., 2022a); (iii) samples of the PlanHab project (*n* = 54) (Šket et al., 2017a,b, 2018, 2020); and (iv) 22 wildcard users (volunteers providing their own .fastq files and necessary metadata; utilized for validation).

Raw sequence files (.fastq files) were downloaded from the EBI (European bioinformatics Institute) next to NCBI Sequence Read Archive and European Nucleotide Archive databases (Gupta et al., 2020) (Supplementary Table S1). Flemish Gut Flora Project data were requested from the Lifelines cohort study^1^ following the prescribed standard protocol for data access. Shotgun sequencing data and metadata are available at the EGA (accession no. EGAS00001003298). Subsequent requests for access to data need to be directed to Flemish Gut Flora consortium.

### 2.2 Sequence data analysis

**Figure 1:**
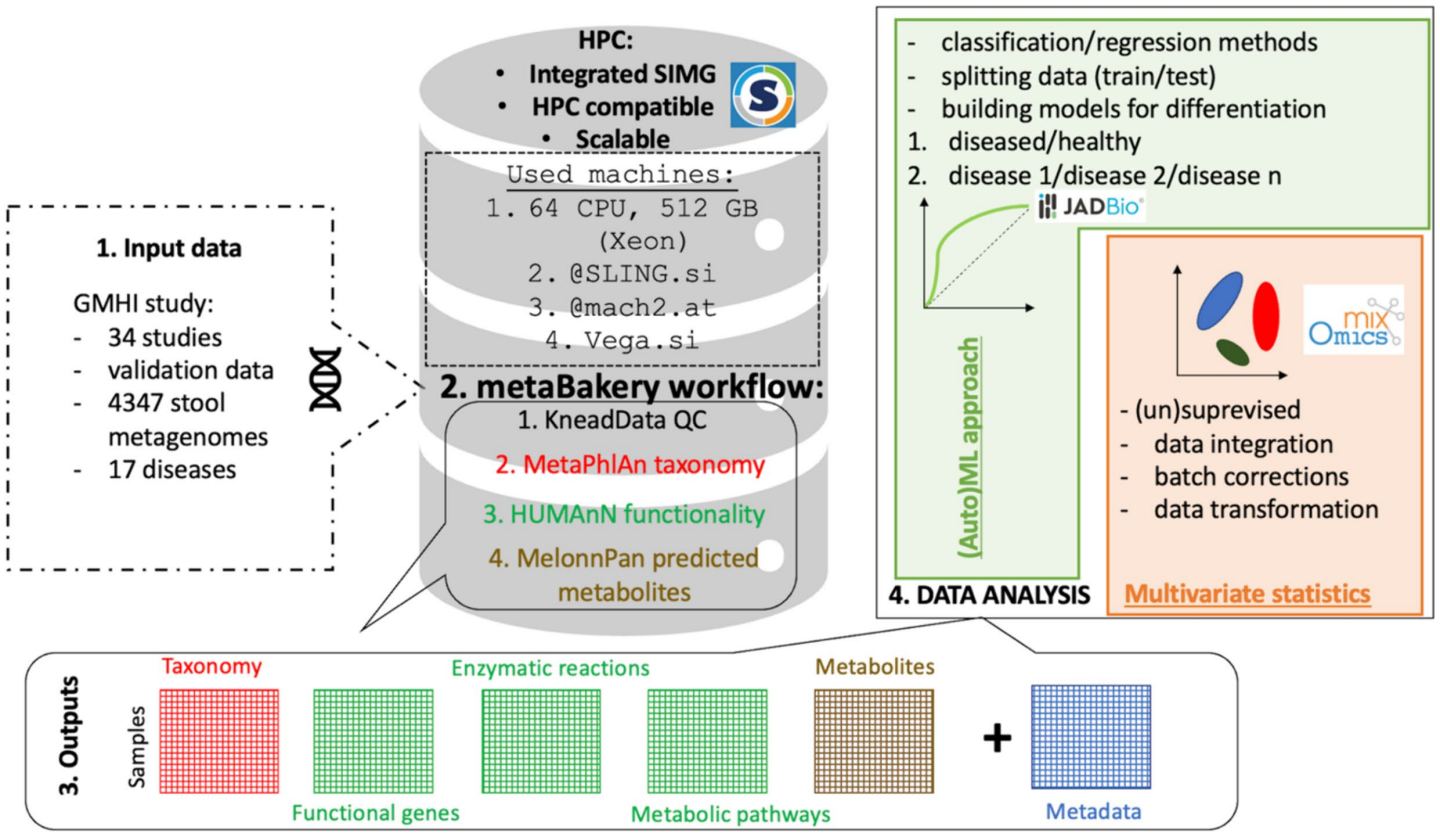
Basic schematic representation of the MetaBakery approach. Highlights: all integrated programs and databases are fully preconfigured; external databases may be used instead of the built-in ones; efficient utilization of computing resources; suitable for autonomous and batch execution; suitable for High-Performance Computing facilities; automatic crash recovery; possibility of splitting large datasets into manageable chunks and processing them separately [on different computers and/or high performance computing (HPC) systems]; transparent handling of paired and unpaired reads (possibly intermixed); transparent handling of major compression formats (.gz, .zip, .bz2, .xz), possibly intermixed; automatic handling of command-line parameters for included programs; experienced users can prescribe custom parameters; efficient restarts with changed parameters and input sets; complete screen and configuration dumping for easy documentation; easy access to command lines, exit codes and messages of programs; versions V4, V3 and V2 of bioBakery programs; only meaningful output files are presented to a user.

All datasets were preprocessed utilizing Slovenian HPC cluster SLING/VEGA infrastructure^2^^,^ ^3^ (accessed 28.2.2024) and Austrian HPC MACH2^4^ (accessed 28.2.2024.) running Singularity-integrated MetaBakery V3. In total, 1.5 million CPU-hours were utilized to perform quality trimming and deconvolute the sequence information into taxonomy, diversity, functional gene, enzymatic reaction and metabolic pathway data layers next to relaxation network predicted metabolites (Figure 1).

In this study we prepared MetaBakery^5^^,^ ^6^ as a skeleton application for a synergistic execution of the bioBakery worklow of programs (McIver et al., 2018)^7^ along with their supporting utilities. Arbitrary number of paired or unpaired fastq files or intermixed serves as input for MetaBakery, either uncompressed or compressed (gzip, zip, bzip2, xz, or mixed) within a single MetaBakery run. The fastq inputs are preprocessed using the KneadData^8^ or skipped for already preprocessed data. The inputs are then subjected to the main analyzing programs: MetaPhlAn (Truong et al., 2015; Blanco-Míguez et al., 2023), HUMAnN (Beghini et al., 2021) and StrainPhlAn (Truong et al., 2015; Beghini et al., 2021) along with their supporting utilities (count feature, regroup table, renorm table and join tables). The original bioBakery functionality was enriched by the integration of MelonnPan (Mallick et al., 2019) for metabolite prediction and Mothur (Schloss et al., 2009) for calculation of microbial alpha diversity. The entire pipeline is executed in a nearly single-click way once input files are put in a directory; a config file may optionally be specified to tailor the execution. The pipeline automatically inspects the computer’s configuration to tune for an efficient execution (Supplementary Figure S1).

The skeleton application within MetaBakery is written in the Python 3 programming language and consists of more than twenty thousand lines of Python code, as well as some utilities written in the C++ programming language for increased efficiency. To achieve efficient running of a number of interdependent programs, an entirely new underlying framework called ExeFlow was developed building from the GUMPP skeleton application (Murovec et al., 2021). To enable its direct adoption for large HPC clusters MetaBakery was packed as Singularity container (Kurtzer et al., 2017; Sochat, 2017; Sochat et al., 2017) to integrate and preconfigure all embedded programs along with their and our own supporting utilities and the relevant databases (Table 1).

**Table 1:** MetaBakery ingredients by its edition enabling comparison of results obtained from various versions of the same utilities.

	MetaBakery V2	MetaBakery V3	MetaBakery V4
Program databases	KneadData 0.12	KneadData 0.12	KneadData 0.12
	human_hg38_RefMrna (default)	human_hg38_RefMrna (default)	human_hg38_RefMrna (default)
	hg37dec_v0.1 (default)	hg37dec_v0.1 (default)	hg37dec_v0.1 (default)
	mouse_C57BL_6NJ	mouse_C57BL_6NJ	mouse_C57BL_6NJ
	SILVA_128_LSUParc_SSUParc_ribosomal_RNA	SILVA_128_LSUParc_SSUParc_ribosomal_RNA	SILVA_128_LSUParc_SSUParc_ribosomal_RNA
Program database	MetaPhlAn 2.7.7	MetaPhlAn 3.1	MetaPhlAn 4.0.6
	v20_m200	v31_CHOCOPhlAn_201901	vJan21_CHOCOPhlAnSGB 202,103
Program databases	HUMAnN 2.8.1	HUMAnN 3.1.1	HUMAnN 3.6.1
	CHOCOPhlAn 0.1.1	CHOCOPhlAn 201901b	CHOCOPhlAn_201901_v31
	UniRef90 1.1 (both, full and EC filtered)	UniRef90 201901b (both, full and EC filtered)	UniRef90 201901b (both, full and EC filtered)
	UniRef50 1.1 (both, full and EC filtered)	UniRef50 201901b (both, full and EC filtered)	UniRef50 201901b (both, full and EC filtered)
Program	StrainPhlAn 1.2.0	StrainPhlAn 3.1.0	StrainPhlAn 4.0.6
Program	MelonnPan	MelonnPan	MelonnPan
Program	Mothur 1.46.1	Mothur 1.46.1	Mothur 1.46.1

Singularity technology was shown to be far better suited for running on high-performance computing facilities compared to other container technologies, like, e.g., Docker (Dirk, 2014) in addition to the fact that it is often the only supported container technology on such large systems.

In addition to improved usability and performance, MetaBakery offers additional benefits (Supplementary Figure S2). The results of all intermediate steps are stored in a specially crafted repository (on a local disk), where each result is associated with its full context, which includes the results of its predecessors and the full set of relevant parameters. On one hand, this enables crash recovery and prompt continuation of processing in the case of a workflow termination (operating system crash, power failure, full hard disk); this feature is offered by the bioBakery (Beghini et al., 2021) workflows as well. In addition, MetaBakery enables efficient re-execution of the workflow with different parameters and/or extended or reduced input data sets. Upon MetaBakery’s re-execution, the available results from an arbitrary number of previous runs are instantly retrieved from the repository. Only new steps are subjected to actual processing. This system opens up the possibility to efficiently experiment with modified parameters or input datasets to observe their effects on the final results. Reuse of the past results is completely automatic and transparent. For example, if after a complete MetaBakery’s run, a user inspects the results and wants to alter some parameters of the HUMAnN step, then results of previous KneadData, MetaPhlAn and StrainPhlAn runs are instantly retrieved from the repository. This does not hold only for the next-to-the-last run, but for an arbitrary number of past runs. In a similar way, subsets of inputs (paired-end or single-end fastq files) may be freely added or removed between different MetaBakery runs, and only the affected processing steps are recalculated.

MetaBakery also provides a crucial feature for processing large human, non-human or environmental metagenomics projects (consisting of hundreds of fastq files or more). Such datasets can only be processed in a reasonable amount of time on HPC platforms. However, HPC policies often prohibit, or at least penalize tasks with long wall times required to process such large input sets. To alleviate this difficulty, MetaBakery provides the ability to split an input dataset into an arbitrary number of subsets (by means of grouping files, not by splitting individual fastq files). The only restriction is that in the case of paired reads, the associated R1.fastq and R2.fastq files remain in the same subset. In the extreme case, each subset may consist of only a single unpaired fastq file or a single R1_R2 fastq pair. These subsets can be processed separately on different computers or HPC nodes, even in different parts of the world. The collected partial results can be subjected to MetaBakery by activating its special mode of operation, in which the final results are reconstructed from the partial ones as if the entire input set had been processed in a single MetaBakery run. The reconstruction consists of all post-processing steps, such us: count feature, regroup table, renorm table and join tables, as well as extended features like Mothur calculations and prediction of metabolites with MelonnPan. In addition to bioBakery enabled databases, a custom built STRUO2 database (Youngblut and Ley, 2021) can be utilized as an external component metaBakery.

MetaBakery is offered in three editions. The first edition contains version 4 of the BioBakery programs (MetaPhlAn 4, HUMAnN 3.6 – to be replaced by version 4 when available, StrainPhlAn 4, along with associated utilities and appropriate databases). The second edition contains version 3 of the BioBakery programs (MetaPhlAn 3, HUMAnN 3, StrainPhlAn 3, with appropriate utilities and databases) (Suzek et al. 2007, 2015). The third edition consists of version 2 of the BioBakery programs (MetaPhlAn V2.7.7, HUMAnN 2.8.1, StrainPhlAn 1.2.0, together with the associated utilities and databases).

In summary, MetaBakery is suitable for standalone execution on both commodity hardware and high-performance computing facilities. All command-line parameters and intermediate file formats are handled automatically by the system, so the end user does not have to deal with these technical details. Nevertheless, experienced users can, if they wish, specify their own parameters for each included program to fine-tune its execution. To facilitate documentation of analyses and subsequent review of executions, MetaBakery stores an exact verbatim copy of its screen output as part of a final report. In addition, the actual command lines, standard output streams (stdout), standard error streams (stderr), and exit codes for each program are stored hierarchically on a disk for ease of navigation, review and debugging. The analysis setup is assisted by optional configuration files, where a complete workflow configuration is prescribed, which also aids in documenting a particular run. All features and mentioned use cases are explained in a user-friendly MetaBakery Users’ Manual^9^ and configuration file template.^10^ MetaBakery highlights are summarized in Table 2.

**Table 2:** MetaBakery highlights.

All integrated programs and databases are fully preconfigured.
External databases may be used instead of the built-in ones (not for V2).
Efficient utilization of computing resources.
Suitable for autonomous and batch execution.
Suitable for High-Performance Computing (HPC) facilities.
Automatic crash recovery.
Possibility of splitting large datasets into manageable chunks and processing them separately (possibly on different computers and/or HPC systems).
Transparent handling of paired and unpaired reads (possibly intermixed).
Transparent handling of major compression formats (gz, zip, bz2, xz), possibly intermixed.
Automatic handling of programs’ command-line parameters.
Experienced users can prescribe custom parameters.
Efficient restarts with changed parameters and input sets.
Complete screen and configuration dumping for easy documentation.
Easy access to command lines, exit codes and messages of programs.
V4, V3 and V2 versions of BioBakery programs.
Only meaningful output files are presented to a user.

The following additional decision steps were taken in analogy with Gupta et al. (2020) when processing datasets with MetaBakery: (i) potential human contamination was filtered by removing reads that aligned to the human genome (reference genome hg19), in addition to repetitive elements; (ii) stool metagenome samples of low read count after quality filtration (<1 M reads) were excluded from our analysis; (iii) the alpha diversity estimates (*n* = 35) were calculated from biome formatted taxonomy profiles in mothur (Schloss et al., 2009). As a result of all the extended additions, MetaBakery acts as re-implementation of the BioBakery workflow (https://huttenhower.sph.harvard.edu/biobakery_workflows/) integrating three versions of tools (V2, V3 and V4) to deliver various microbiome layers of information: (i) taxonomy (Bacteria, Archaea, Fungi, Protozoa, and Viruses), (ii) alpha diversity estimates; (iii) functional genes, (iv) enzymatic reactions, (v) metabolic pathways, and (vi) predicted metabolites, that are utilized next to subject (patient or healthy) metadata.

### 2.3 Data content

The entire pipeline was used on two different datasets focusing on human microbiome studies: (i) smaller dataset [depression data; [(Valles-Colomer et al., 2019); accession no. EGAS00001003298] consisting of *n* = 80 samples from patients with depression and *n* = 70 healthy controls] and (ii) larger dataset (*n* = 4,976 samples - healthy controls and patients with different diseases such as ACVD, ankylosing spondylitis, colorectal adenoma, colorectal cancer, Crohn’s disease, impaired glucose tolerance, IBD, obesity, liver cirrhisos, NAFLD, overweight, rheumatoid arthiritis, type 2 diabetes, symptomatic atherosclerosis, ulcerative colitis and underweight) (Gupta et al., 2020; Deutsch et al., 2022a). Both datasets were previously published in scientific journals to ensure the comparability and efficiency of the MetaBakery tool.

In total, 4,976 samples were processed in this study within 1.5 mio CPU-hours at SLING/VEGA HPC cluster^11^ (accessed 28.2.2024).

The resulting six data matrices (taxonomy, diversity, functional genes, enzymatic reactions, metabolic pathways and predicted metabolites) were matched with the corresponding human subject metadata matrix and prepared for subsequent machine learning step.

The analyses were run on complete data. Sequences for 4,976 individuals with different diseases and healthy cohorts as control group were downloaded. Bioinformatics was completed with our Singularity implemented pipeline and produced the following information tables: (i) taxonomy table (2,408 variables, file size 0.03 Gb); (ii) gene families (11,451,445 variables, file size 134 Gb); (iii) enzymatic reactions (622,447 variables, file size 8 Gb); (iv) metabolic pathways (47,536 variables, file size 0.6 Gb); (v) predicted metabolites (80 variables, 0.008 Gb); (vi) diversity estimates (35 variables, file size 0.005 Gb); (vii) participant metadata (10 variables, 0.003 Gb).

The compilation of all these variables for almost 5,000 samples produced a matrix with 13 million rows, exhibiting all of the characteristics of microbiome data (Marcos-Zambrano et al., 2021, 2023; Moreno-Indias et al., 2021; Ibrahimi et al., 2023; Papoutsoglou et al., 2023). Contrary to previous approaches (Gupta et al., 2020; Su et al., 2022) that involved significant data reduction steps using arbitrary assumptions (i.e., average OTU abundance <0.15, prevalence >5%) we did not involve such steps as there is no previous guidance on how to set the values in other information layers (diversity, functional gene, enzymatic reactions, metabolic pathways, predicted metabolites) or whether the same settings are transferable between information layers or which variables represent noise within or between multiclass categories.

Benjamini–Hochberg correction was used to control for multiple testing, and results were considered significant at false discovery rate (FDR) < 0.05 as described before in our past studies (Šket et al., 2017a,b, 2018, 2020; Murovec et al., 2020, 2021; Deutsch et al., 2021, 2022a,b; Deutsch and Stres, 2021).

### 2.4 Machine learning

Automated machine learning, Just Add Data Bio (JADBio), an Amazon cloud based machine learning platform for analyzing potential biomarkers (Tsamardinos et al., 2022), was used to search for biomarkers on both datasets. The JADBIO platform was developed for predictive modeling and providing high-quality predictive models for diagnostics using state-of-the-art statistical and machine learning methods. Personal analytic biases and methodological statistical errors were eliminated from the analysis by autonomously exploring different settings in the modeling steps, resulting in more convincing discovered features to distinguish between different groups. JADBIO with extensive tuning effort and six CPUs was used to model different dataset choices in addition to the features observed in samples of all groups from different projects by splitting the total data into a training set and a test set in a 70:30 ratio. The training set was used to train the model and the test set was used to evaluate the model (Deutsch et al., 2022a). The modeling step was evaluated using 12 different performance metrics (AUC, mean average precision, accuracy, F1 score, Matthews correlation, precision, true-positive rate, specificity, true-positive, true-negative, false-positive, and false-negative). In all cases, 10-fold cross-validation without drop (with a maximum of 20 repeats) was performed. 1,000–3,000 different model configurations (with different feature selection and predictive algorithms with different hyperparameters) were used and up to 100,000 different models were trained per each of the six datasets. The largest dataset representing the gene family data set was reduced to obtain rows with less than 25% zeros per row.

## 3 Results and discussion

### 3.1 MetaBakery development, streamlining and large-scale utilization

MetaBakery represents an integrated ready-made system that shortcuts the nontrivial need for technical details of installing and configuring the included programs, libraries and databases. Nevertheless, the high level of flexibility is retained as the integrated databases can be freely substituted by advanced users, amended with configuration setting options available to them^12^^,^ ^13^ (Schloss et al., 2009; Segata et al., 2012; Truong et al., 2015; Pasolli et al., 2017; Franzosa et al., 2018; McIver et al., 2018; Mallick et al., 2019; Schloss, 2020; Beghini et al., 2021).

The pipeline handles parallelism differently than the bioBakery as CPUs are always allocated to all running tasks guided by performance parameters (determined by empirical measurements in this study) that indicate the use of CPUs and disk by individual programs to execute as many tasks as possible in parallel without overloading the underlying hardware. Single-threaded or less efficiently parallelized programs no longer take up an entire group of CPUs for themselves, since they are executed evenly on all CPUs in parallel with other processing steps. Better resource utilization thus results from the simultaneous execution of multiple programs on the same set of CPUs which is of special importance when dealing with short HPC wall times. The built-in performance parameters are fully configurable although MetaBakery’s default settings were determined by empirical measurements on various pieces of hardware: (i) HPC nodes with varying numbers of CPUs from 256 down to 16, (ii) a desktop computer with dual XEON processor with 64 hyper-threaded processors, and (iii) less powerful desktop computers with 12 and 8 CPUs. Hence, based on the test results our MetaBakery was programmed to tune itself to perform out-of-the-box on the entire hardware spectrum (Supplementary Figure S2).

MetaBakery is offered in three editions. The first edition contains version 4 of the BioBakery programs (MetaPhlAn 4, HUMAnN 3.6 – to be replaced by version 4 when available, StrainPhlAn 4, along with associated utilities and appropriate databases). The second edition contains version 3 of the BioBakery programs (MetaPhlAn 3, HUMAnN 3, StrainPhlAn 3, with appropriate utilities and databases). The third edition consists of version 2 of the BioBakery programs (MetaPhlAn V2.7.7, HUMAnN 2.8.1, StrainPhlAn 1.2.0, together with the associated utilities and databases).

### 3.2 Large scale computing results: 4976 taxonomy layers

**Figure 2:**
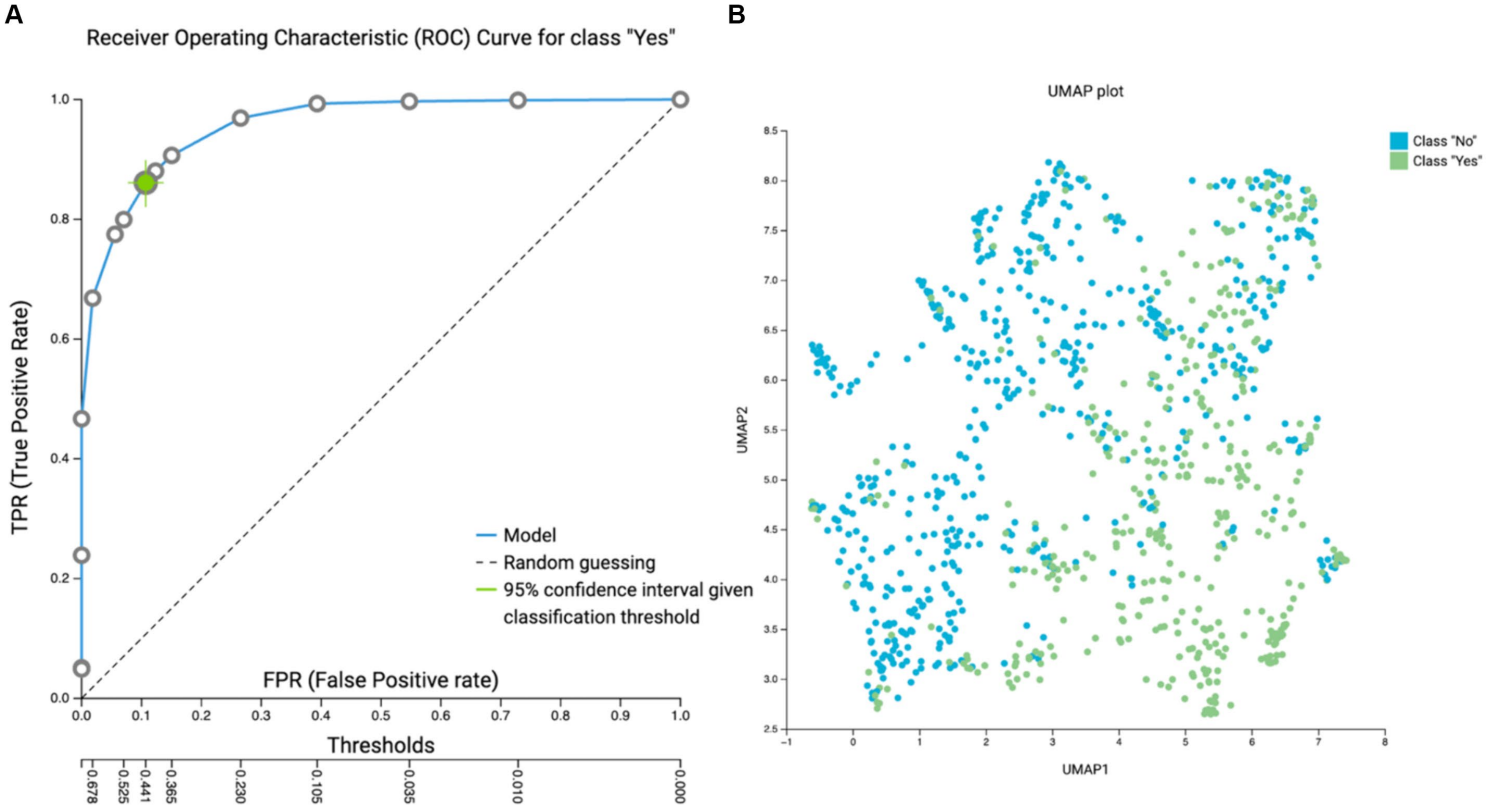
**(A)** Receiver operating characteristic (ROC) curve (AUC = 0.959) for class “Yes” = diseased. **(B)** Uniform manifold approximation and projection (UMAP) attempts to learn the high-dimensional manifold on which the original data lays, and then maps it down to two dimensions. UMAP plots provides a visual aid for assessing relationships among samples.

Our data integration resulted in utilization of 4,976 samples encompassing healthy and 16 disease states from 35 studies of 15 countries. In our first data analysis we focused on delineation between the two groups, namely the healthy on one side and a group of disease states on the other. Overall taxonomy classification efficiency enabled us to build a relatively simple and effective model without any specific filtering as also deployed before in the past studies (Gupta et al., 2020) based on taxonomy information only. In essence, we were able to utilize taxonomy information to clearly separate healthy from the diseased states (Figure 2 and Supplementary Figure S3).

**Figure 3:**
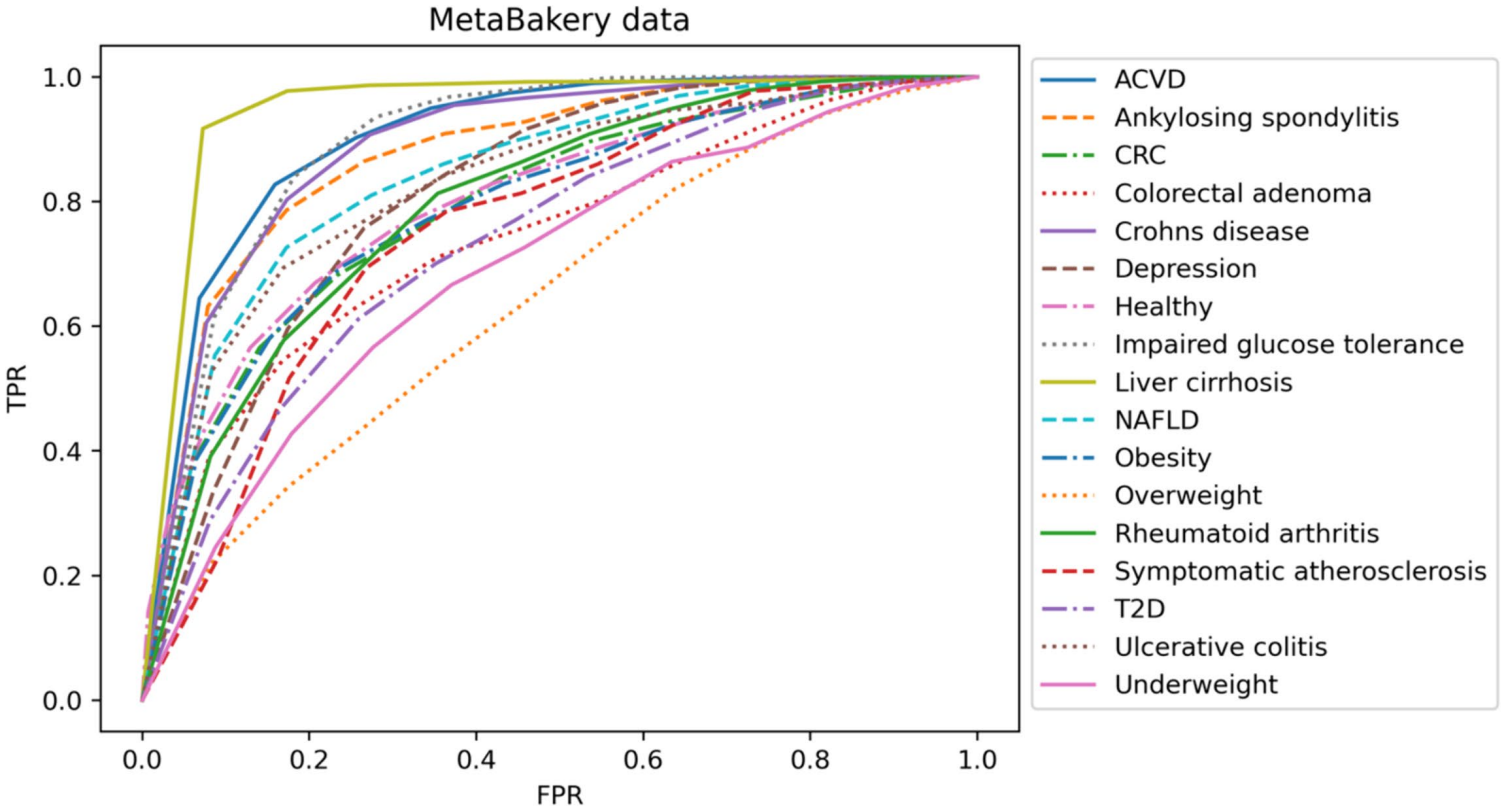
Representation of multiclass classification based on AUC curves utilizing taxonomy data layer only and different diseases. TPR, true positive rate; FPR, false positive rate.

In our second analysis we focused on multiclass problem of distinguishing various disease states among themselves. Classification models for many of the disease states based on taxonomy only utilizing rather modest numbers of samples also showed the clear need for larger cohorts on the one side, however clearly provided the necessary information that the signal can readily be detected in such small size data as well, guiding future larger-scale data integration (Figure 3).

Diversity metrics utilizing 35 indices were integrated as one of the outputs of the MetaBakery pipeline. For this purpose, the standard diversity calculators from Mothur (Schloss et al., 2009) were integrated into the MetaBakery pipeline, which combine the entire analytical concept of modern microbiology in one pipeline (Supplementary Figure S4), extending the so far amplicon centered approach to metagenomics in a streamlined way.

### 3.3 Large scale computing results: depression dataset

In our third analysis we focused on depression dataset, utilizing data integration of taxonomy, diversity, functional genes, enzymatic reactions, metabolic pathways and metabolites. Overall, variables were tested for information content that would separate healthy from the clinically depressed participants. We took a two-step approach to model the depression data. In the first step, taxonomy data (852 variables), gene family data (596,146 variables), enzymatic reactions (237,025 variables), metabolic pathways (14,525 variables), and predicted metabolites (80 variables) were modeled individually. In the second step, only the most important features were then modeled on the merged dataset (97 variables). In addition, taxonomy data from 3 different MetaPhlAn versions were also modeled (MetaPhlAn 2.0–972 variables, MetaPhlAn 3.0–859 variables, and MetaPhlAn 4.0–4,249 variables) (Supplementary Table S2). A binary classification was used to distinguish between healthy and depressed individuals.

**Figure 4:**
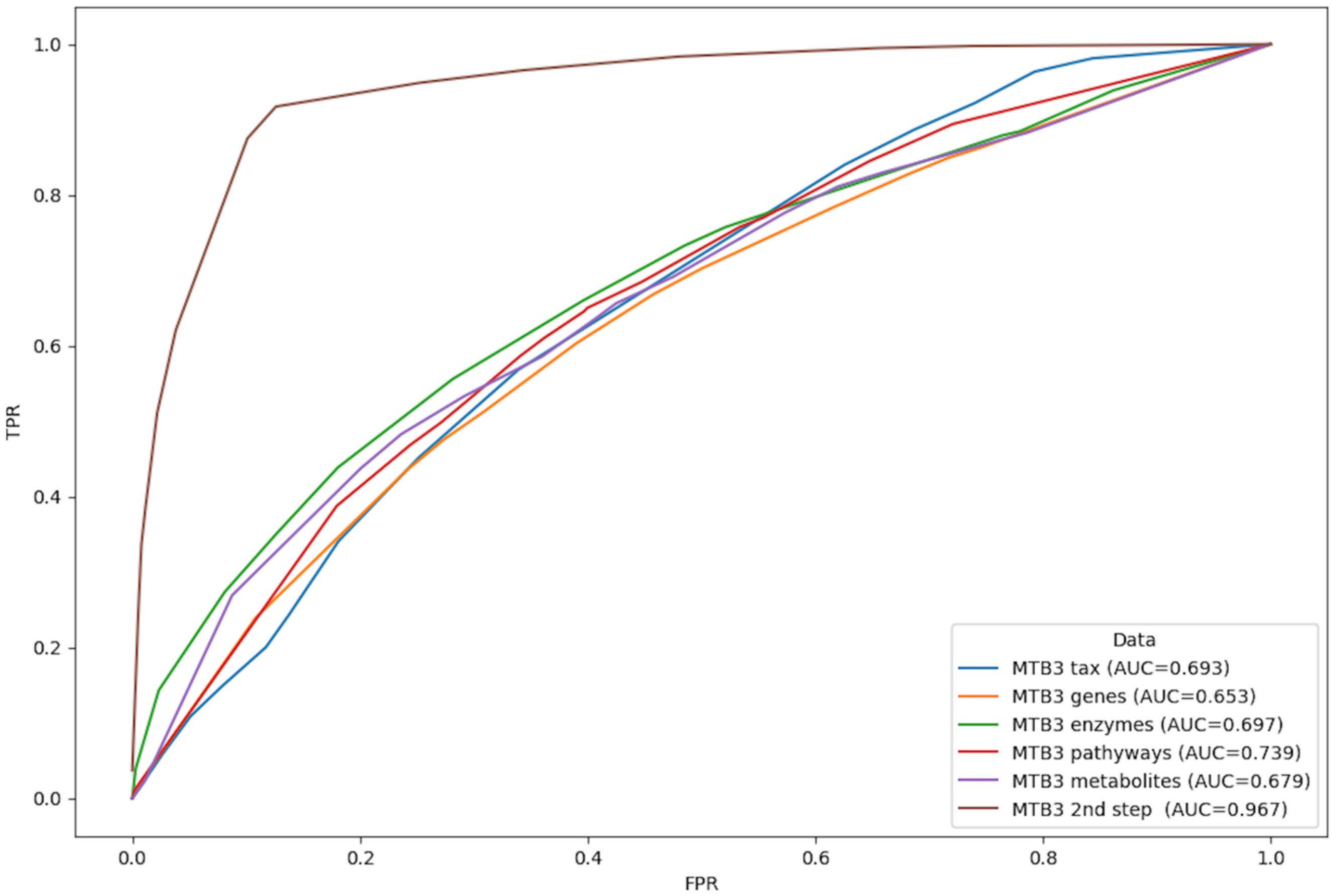
Representation of classification based on AUC curves between healthy individuals and patients with depression based on individual information layers: taxonomy (blue), functional genes (orange), enzymatic reactions (green), metabolic pathways (red) and predicted metabolites (purple) calculated with MetaBakery3. Brown line represents the most successful model utilizing the collected features detected as the most important in all data matrices in one analysis. TPR, true positive rate; FPR, false positive rate.

At the taxonomy level, 23 features (MetaBakery version 2.0), 22 features (Metbakery version 3.0), and 25 features (Metbakery version 4.0) were found to be the most significant in distinguishing depression patients from healthy individuals (Supplementary Figure S5). Because the AUC was highest in MetaBakery 3.0, the corresponding functional data were used to build more successful models at the functional fingerprint level (gene families, enzymatic reactions, metabolic pathways, predicted metabolites). Nine genes, 25 enzymatic reactions, 16 metabolic pathways, and 25 predicted metabolites were discovered in each corresponding data set using JADBio ML (Supplementary Figure S6). In the last step, a subset of the significant features from the first step was used to improve the model. And the logistic ridge model with an AUC of 0.967 was constructed to distinguish patients with depression from healthy individuals (Figure 4).

## 4 Conclusion

In this study, we presented MetaBakery,^14^ an integrated application designed as a framework for synergistically executing the bioBakery workflow (Franzosa et al., 2018; McIver et al., 2018; Beghini et al., 2021) and associated utilities. MetaBakery streamlines the processing of any number of paired or unpaired fastq files, or a mixture of both, with optional compression (gzip, zip, bzip2, xz, or mixed) within a single run. MetaBakery uses programs such as KneadData,^15^ MetaPhlAn, HUMAnN and StrainPhlAn as well as integrated utilities and extends the original functionality of bioBakery. In particular, it includes MelonnPan for the prediction of metabolites and Mothur for calculation of microbial alpha diversity. Written in Python 3 and C++, this near single-click pipeline encapsulated as Singularity container leverages the ExeFlow framework for efficient execution on various computing infrastructures, including large High-Performance Computing (HPC) clusters. MetaBakery facilitates crash recovery, efficient re-execution upon parameter changes, and processing of large data sets through subset handling. MetaBakery is offered in three editions with bioBakery ingredients versions 4, 3 and 2. MetaBakery is versatile, transparent and well documented, with functions described in the MetaBakery Users’ Manual.^16^ It provides automatic handling of command line parameters, file formats and comprehensive hierarchical storage of output to simplify navigation and debugging. MetaBakery filters out potential human contamination and excludes samples with low read counts. It calculates estimates of alpha diversity and represents a comprehensive and augmented re-implementation of the bioBakery workflow. The robustness and flexibility of the system enables efficient exploration of changing parameters and input datasets, increasing its utility for microbiome analysis. Furthermore, we have shown that MetaBakery tool can be used in modern biostatistical and machine learning approaches including large-scale microbiome studies, potentially providing completely new insights into the microbial world.

## Data availability statement

The datasets presented in this study can be found in online repositories. The names of the repository/repositories and accession number(s) can be found in the article/Supplementary material.

## Ethics statement

Ethical approval was not required for the study involving humans in accordance with the local legislation and institutional requirements. Written informed consent to participate in this study was not required from the participants or the participants’ legal guardians/next of kin in accordance with the national legislation and the institutional requirements.

## Author contributions

BM: Writing – original draft, Writing – review & editing, Data curation, Formal analysis, Methodology, Software. LD: Writing – original draft, Writing – review & editing, Data curation, Formal analysis, Investigation, Methodology, Software, Validation. DO: Writing – original draft, Writing – review & editing, Conceptualization, Funding acquisition, Resources. BS: Writing – original draft, Writing – review & editing, Conceptualization, Data curation, Formal analysis, Funding acquisition, Investigation, Methodology, Project administration, Resources, Software, Supervision, Validation, Visualization.

## Footnotes

^1^ https://lifelines.nl/lifelines-research/access-to-lifelines

^2^ https://en-vegadocs.vega.izum.si/

^3^ https://www.sling.si/en/sling-2/

^4^ https://www.uibk.ac.at/zid/systeme/hpc-systeme/mach2/

^5^ http://metabakery.fe.uni-lj.si

^6^ http://metabakery.fe.uni-lj.si/metabakery_manual.pdf

^7^ https://huttenhower.sph.harvard.edu/biobakery_workflows/

^8^ https://github.com/bioBakery/kneaddata

^9^ http://metabakery.fe.uni-lj.si/metabakery_manual.pdf

^10^ http://metabakery.fe.uni-lj.si/config_template.txt

^11^ https://en-vegadocs.vega.izum.si/

^12^ http://metabakery.fe.uni-lj.si/metabakery_manual.pdf

^13^ http://metabakery.fe.uni-lj.si/config_template.txt

^14^ http://metabakery.fe.uni-lj.si

^15^ https://github.com/bioBakery/kneaddata

^16^ http://metabakery.fe.uni-lj.si/metabakery_manual.pdf
