# Supplementary material for: MetaBakery: a Singularity implementation of bioBakery tools as a skeleton application for efficient HPC deconvolution of microbiome metagenomic sequencing data to machine learning ready information
Source: Front Microbiol. 2024 Jul 30;15:1426465. doi: 10.3389/fmicb.2024.1426465 (PMC11321593; doi:10.3389/fmicb.2024.1426465)
Supplement: Supplementary file 2 [file Table_2.DOCX]

Supplementary Material

MetaBakery: a Singularity implementation of bioBakery tools as skeleton application for efficient HPC deconvolution of microbiome metagenomic sequencing data to machine learning ready information

Boštjan Murovec^1^, Leon Deutsch^2,3^, JADBio^4^, Damjan Osredkar^5,6^, Blaž Stres^2,7,8,9*^

^1^ University of Ljubljana, Faculty of Electrical Engineering, Ljubljana, Slovenia

^2^ Department of Animal Science, Biotechnical faculty, University of Ljubljana, Ljubljana, Slovenia

^3^ The NU, The NU B.V., Leiden, The Netherlands

^4^Jadbio

^4^Damjan

^4^Damjan

^7^D13 Department of Catalysis and Chemical reaction Engineering, National Institute of Chemistry, Ljubljana, Slovenia

^8^Institute of Sanitary Engineering, Faculty of Civil and Geodetic Engineering, Ljubljana, Slovenia

^9^Department of Automation, Biocybernetics and Robotics, Jožef Stefan Institute, Ljubljana, Slovenia


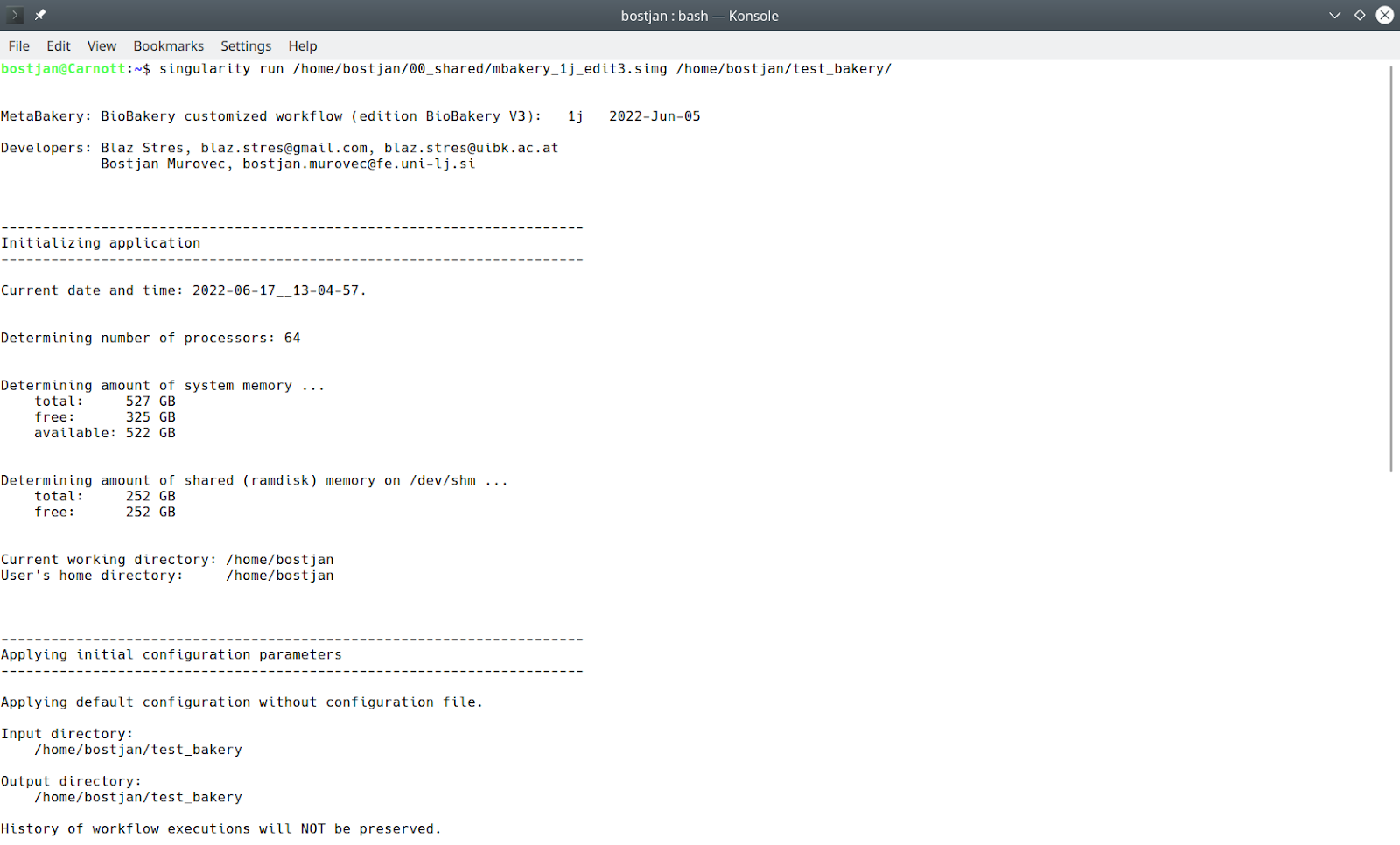


**Supplementary Figure 1.** Start of MetaBakery execution.


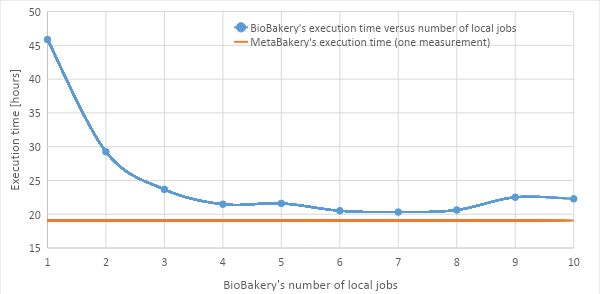


**Supplementary Figure 2.** BioBakery and MetaBakery performance comparison; MetaBakery is self tuned.


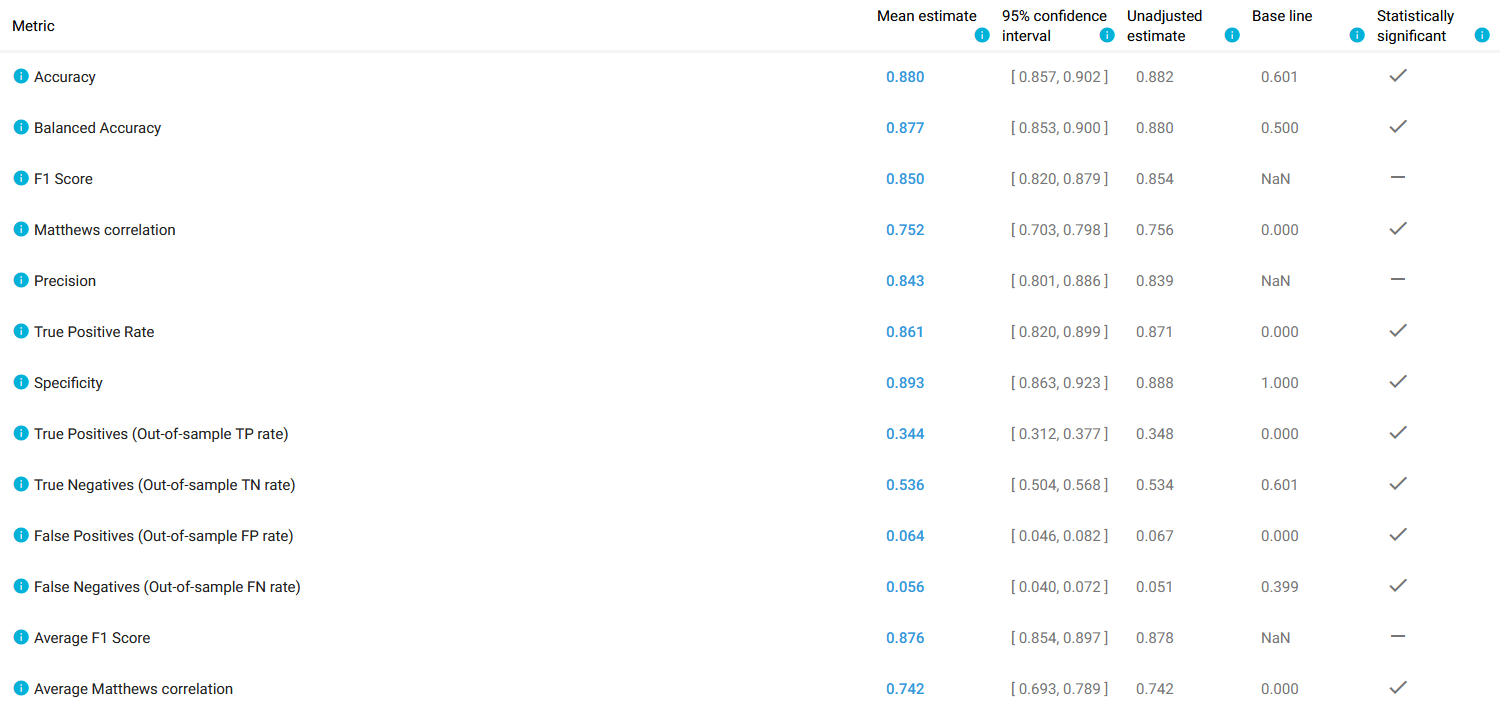


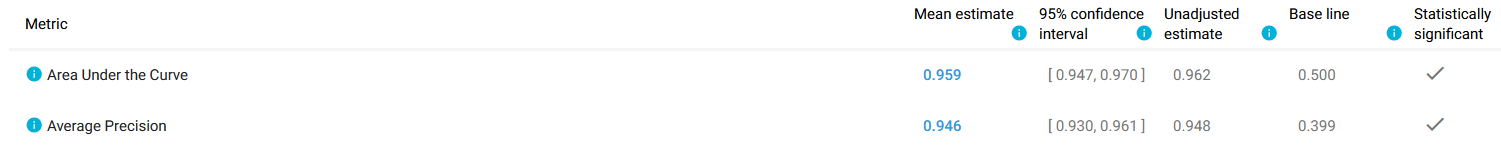
**Supplementary Figure 3.** An example of various descriptive metrics illustrating the performance of JADBio machine learning on GMHI taxonomy dataset.


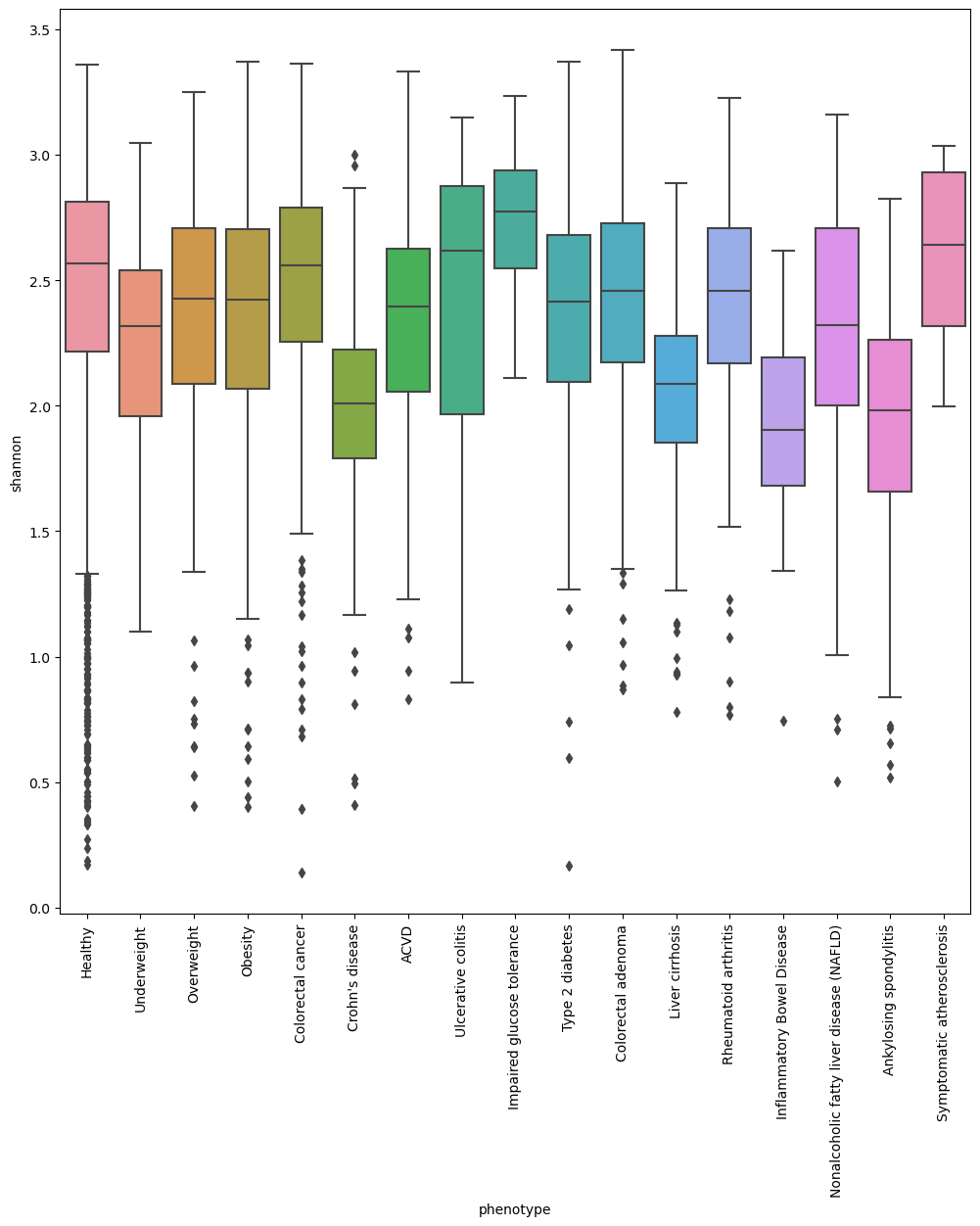


**Supplementary Figure 4.** Shannon diversity across all disease in large dataset calculated with MetaBakery. Numerous diversity indices can be calculated using the standard approaches within the program Mothur that was inbuilt into the MetaBakery presented in this work.


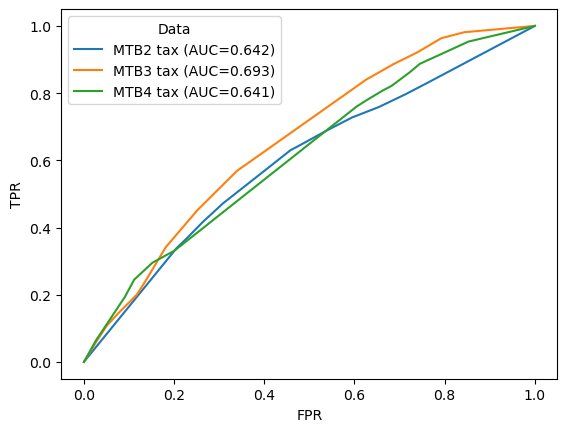


**Supplementary Figure 5.** Performance of JADBio three different versions (MetaBakery2, Metabakery3, MetaBakery4) on taxonomy data on depression dataset. TPR – True Prediction Rate; FPR – False Prediction Rate.


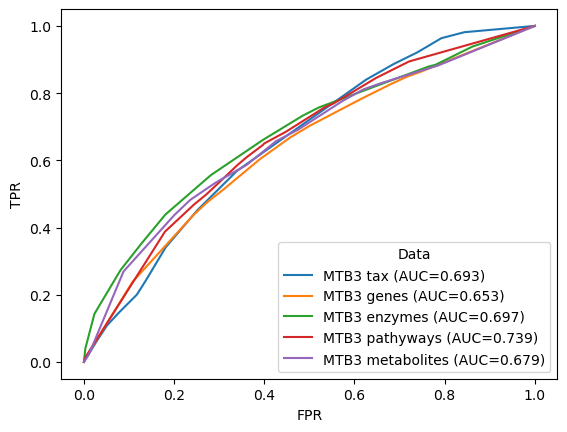


**Supplementary Figure 6.** Performance of JADBio machine learning on the separate matrices from the microbiome information layers produced with MetaBakery3. TPR – True Prediction Rate; FPR – False Prediction Rate.
